# The Immune Response to *Trypanosoma cruzi*: Role of Toll-Like Receptors and Perspectives for Vaccine Development

**DOI:** 10.1155/2012/507874

**Published:** 2012-02-16

**Authors:** Mauricio M. Rodrigues, Ana Carolina Oliveira, Maria Bellio

**Affiliations:** ^1^Centro de Terapia Celular e Molecular (CTCMol), Universidade Federal de São Paulo (UNIFESP), 04044-010 São Paulo, SP, Brazil; ^2^Instituto de Biofísica Carlos Chagas Filho, Universidade Federal do Rio de Janeiro (UFRJ), 21941-902 Rio de Janeiro, RJ, Brazil; ^3^Instituto de Microbiologia Paulo de Góes, Centro de Ciências da Saúde, Universidade Federal do Rio de Janeiro (UFRJ), CCS, Avenida Carlos Chagas Filho, 373 Bloco D, sala 35, Cidade Universitária, 21941-902 Rio de Janeiro, RJ, Brazil

## Abstract

In the past ten years, studies have shown the recognition of *Trypanosoma cruzi*-associated molecular patterns by members of the Toll-like receptor (TLR) family and demonstrated the crucial participation of different TLRs during the experimental infection with this parasite. In the present review, we will focus on the role of TLR-activated pathways in the modulation of both innate and acquired immune responses to *T. cruzi* infection, as well as discuss the state of the art of vaccine research and development against the causative agent of Chagas disease (or American trypanosomiasis).

## 1. Introduction

*Trypanosoma cruzi* is an intracellular trypanosomatid protozoan, which is transmitted to the human host by blood-feeding reduviid bugs, members of the insect subfamily Triatominae. Other modes of transmission include oral infection through contaminated food, congenital transmission, blood transfusions, organ transplants, and by accidental laboratory inoculation. This parasite, as well as its vector and the disease it causes, was first described by Chagas in 1909 [1]. Presently, the World Health Organization (WHO) estimates that approximately 10 million people are infected [2]. While Chagas disease is endemic to Central and South America, in the last years infected individuals have also been registered among immigrants in the United States, Europe, and Japan [3]. Although most of these cases were imported from the endemic regions, vector-transmitted autochthonous infections have also been documented in the United States. This fact and the lack of mandatory screening for all blood and tissue donors point to a possible altered epidemiology of Chagas disease in a near future.

 The determinants of Chagas disease come from the burden and the lineage of the inoculated parasite, as well as the infection route and the immune competent status of the host. Two different phases of the disease follow the entrance of *T. cruzi* into the host (for a review see [4]). The acute phase lasts around two months and is asymptomatic in most infected individuals although some patients can present symptoms like prolonged fever, anorexia, nausea, vomiting, and diarrhea. During this phase, high numbers of parasites are frequently found in the host bloodstream and tissues, as well as high plasma levels of cytokines and intense activation of B and T lymphocytes. Also, lymphoadenopathy, splenomegaly, and intense inflammatory processes may be associated with parasite nests within tissues. A small percentage (5–10%) of infected individuals can develop a more severe condition, presenting myocarditis or meningoencephalitis, which they may die of. Most of the contaminated individuals remain asymptomatic (indeterminate form) often for years or even decades, but, then, around 30% of patients develop cardiac or gastrointestinal complications, characteristics of the chronic phase of the Chagas disease. The pathological basis of chronic chagasic cardiomyopathy (CCC) has been a matter of intense debate for years. Immunopathology due to parasite persistence is considered a key element in the development of CCC although there is also evidence of a role for autoimmunity. During the chronic phase (indeterminate or not) few or no parasites are found in the circulation, but reactivation may occur by immunosuppression, particularly AIDS, and by pregnancy. The only effective and approved drugs in the treatment of the acute phase, or of the reactivation of the disease, are nitrofuran (nifurtimox, Lampit) and nitroimidazole (benznidazole, Rochagan), which are not fully satisfactory because of their limited efficacy in the chronic stage and of their important adverse side effects. Host control of *T. cruzi *has been shown to depend on both humoral and cell-mediated adaptive responses as well as on elements of the innate immune system [5]. To date, however, no human vaccine against infection with *T. cruzi* is currently available. Finally, the economic and social burdens due to early morbidity and mortality caused by Chagas disease are considerable, leading to high economic losses in Latin America. Understanding of the pathogenesis of Chagas disease will add to the development of new molecular targets for prophylactic vaccines and drug therapies, which are of extreme need for combating this emerging neglected disease.

## 2. Innate Immunity and TLRs

For a long time innate responses were believed to be nonspecific to the invading pathogen. In contrast, acquired immunity mediated by T and B lymphocytes was shown to display a fine specificity for the different pathogen-derived antigens through the employment of clonal receptors, which result from the genetic recombination of hundreds of different gene segments. The discovery in 1996 that the *Drosophila* transmembrane protein Toll specifically mediates the recognition and the response to fungal infection [6], followed by the cloning of several related receptors in other species, including human [7] and the discovery that one of these molecules (TLR4) is the receptor for lipopolysaccharide (LPS) [8], challenged the dogma that attributed nonspecificity to innate immunity. Owing to the new receptors' similarity to the Drosophila Toll, these molecules were called Toll-like receptors, or TLRs. So far, 10 and 12 different functional TLR-family members have been identified in man and mice, respectively, of which TLRs 1–9 are conserved in both species, TLR10 is selectively expressed in humans and TLR11, TLR12 and TLR13 are present in mice but not in humans (reviewed in [9]). Each TLR recognizes different chemical structures, which are highly conserved in microorganisms and collectively referred to as pathogen-associated molecular patterns (PAMPs). Among these are lipids, carbohydrates, nucleic acids, and various proteins derived from bacteria, viruses, fungi, protozoa, and helminth parasites. Moreover, TLR-signaling pathways may also be activated by self components released by tissue damage or inflammation, the so-called damage-associated molecular patterns (DAMPs), which alert the immune system to danger resulting either from sterile insult or from infection [10]. To learn the detailed mechanisms by which the innate immune system detects and responds to parasites is crucial to understanding how infection is controlled. However, only recently insights into how the TLR-signaling system responds to infection by protozoans, including *Trypanosoma cruzi, Trypanosoma brucei, Leishmania *spp., *Plasmodium *spp*. and Toxoplasma gondii* have emerged [11]. Different TLRs show a diverse expression pattern in a variety of cells and tissues, as well as different subcellular localization (either on the cell surface or within endosomal compartments). Although a certain degree of redundancy exists between signals induced by the various TLRs, recent studies have identified signaling pathways specific for individual TLRs, involving different adaptor molecules responsible for signal transduction. This leads to cytokine release profiles specific for particular PAMPs, and, thus, TLRs confer a certain degree of specificity to the innate-immune response. The formation of heterodimers among diverse TLRs (as TLR2/TLR6 or TLR2/TLR1) or the employment of accessory molecules (as CD14 or CD36), for the recognition of certain PAMPs but not others, creates a further degree of specificity [12]. Recognition of microbial components by TLRs triggers the initial innate immune response leading to inflammatory gene expression and, eventually, to the clearance of the infectious agent. Moreover, TLR-mediated recognition, by inducing the maturation of dendritic cells and, consequently, directing the T helper responses, represents a link between the innate- and acquired-immune systems [9]. Finally, as a result of studies searching for TLR agonists and antagonists, as well as for inhibitors of TLR-signaling pathways, drugs with these properties are currently being tested in a variety of therapeutic applications, and at least one TLR agonist (monophosphoryllipid A-MPL) has already been approved as adjuvant in vaccines [13].

 Other germline-encoded innate receptor families were discovered in the last years and, together with TLRs, are collectively called pattern-recognition receptors (PRRs). These include membrane-bound C-type lectin receptors (CLRs), cytosolic proteins such as nucleotide-binding oligomerization domain (NOD)-like receptors (NLRs), and RIG-I-like receptors (RLRs) (reviewed in [14]). Although TLRs play a central role in the initiation of immune responses against different pathogens, microbes display multiple PAMPs, which activate both TLRs and other PRRs, becoming evident that PRRs other than TLRs are also involved in the control of innate immunity. Moreover, while TLR ligand specificity, signaling pathways, and cellular trafficking have been broadly studied, less is known about the expected crosstalk between different PRR pathways, and the consequences that such an interaction would have for the induction of effective innate and acquired immune responses.

 As reviewed here, after infection with *T. cruzi,* several inflammatory genes are activated through different TLR pathways. This leads to inflammatory response and induction of diverse effector mechanism of the adaptive immune response, which culminates with pathogen control, though the sterile cure is not achieved. On the other hand, very little is known about *T. cruzi *recognition by other PRRs. Recently, the first example of NLR-dependent response accounting for host resistance against infection with a protozoan has been reported [15]. In this work, *Nod1^−/−^* mice were shown to be very susceptible to *T. cruzi,* succumbing to the infection and displaying higher parasitemia and parasite loads in the spleen and heart tissues, although NOD1 deficiency does not impair the production of different cytokines as IL-12, TNF-*α*, IFN-*γ*, or IL-10. As *T. cruzi* parasites lack peptidoglycan or any known agonist for NOD1, it would be interesting to determine whether NOD1 directly senses a *T. cruzi*-derived PAMP, or if the NOD1 pathway is indirectly activated during infection. Therefore, the detailed mechanism by which NOD1 confers resistance to infection with *T. cruzi *remains to be described and a possible cross-talk between NLR and TLR pathways during infection with* T. cruzi* waits for further investigation.

## 3. TLR Agonists Expressed by *T. cruzi*

In the past years, different groups have identified diverse *T. cruzi*-derived molecules that act as TLR agonists, inducing the production of nitric oxide (NO) and the secretion of inflammatory cytokines and chemokines by cells of the monocytic lineage. The first major class of *T. cruzi* molecules to be characterized as PAMPs was trypomastigote-derived glycosylphosphatidylinositol (tGPI) anchors of mucin-like glycoproteins, which are distributed at the cell-surface membrane of *T. cruzi* and were identified as potent activators of TLR2 from both mouse and human origin [16]. Proinflammatory activity of tGPI was shown to be dependent on its fine structure, mainly the unsaturated fatty acid at the sn-2 position of the alkylacylglycerolipid component. In contrast, another member of the GPI family purified from epimastigote forms, named glycoinositolphospholipid (eGIPL) and whose lipid moiety is instead composed by a *N*-lignoceroylsphinganine, was shown to induce NF-*κ*B activation via TLR4 [17]. GIPLs are free anchors abundantly present at the surface membrane of all parasite stage forms, presenting different biological effects on different cell types [18, 19]. Importantly, the structure of GIPLs displayed by the infective metacyclic trypomastigote and by the epimastigote forms is very similar to each other, containing the same conserved Man4-GlcN glycan sequence and the myo-inositol-phosphate-lipid moiety predominantly (70%) formed by inositol-phosphoceramides, although its constitution may change depending on the *T. cruzi *strain [20]. For example, while GIPLs from Y, G, and Tulahuen strains contain ceramide, those from the CL strain are a mixture of dihydroceramide and alkylacylglycerol species [21]. Therefore, the variable lipid moiety composition of different GPI anchors determines whether their recognition is mediated by TLR2 (alkylacylglycerol) or TLR4 (dihydroceramide). Although tGPI (TLR2 agonist) and eGIPL from Y strain (TLR4 agonist) were not compared in the same assay for their relative capacity of inducing proinflammatory responses on cells expressing normal levels of TLR2 and TLR4 molecules, results obtained with human TLR2-transfected CHO cells, which also express endogenous levels of hamster TLR4, suggested a 100-fold superior activity of tGPI anchors [16]. An interesting point yet to be investigated is whether these different GPI anchors, which may be released by the parasite by shedding [22] and whose inflammatory activity depends on TLR2 or TLR4, present synergistic properties. Of note, genome-wise prediction analysis revealed that approximately 12% of *T. cruzi* genes possibly encode GPI-anchored proteins, a number much higher than in previously studied protozoa or mammalian species [23]. Moreover, the recent large-scale analysis of GPI-anchored molecules identified 78 GIPLs and 11 ptn-GPIs, of which 70 GIPLs and 8 ptn-GPIs were not previously described [23]. Among these, probably novel TLR2 and/or TLR4 agonists will be characterized. Other differences between *T. cruzi*-derived GIPL and tGPI anchors were determined concerning the participation of coreceptor molecules on their recognition and the triggered signaling pathway. For instance, while anti-CD14 antibodies blocked the production of TNF-*α* by human macrophages exposed to tGPI-mucin *in vitro* [24], neutrophil attraction to the peritoneal cavity triggered by the injection of eGIPL was maintained in CD14-deficient mice, indicating that eGIPL is recognized by TLR4 in a CD14-independent way (Bellio, M., unpublished results). Also, TNF-*α* and MIP-2 production in response to GIPL was shown to be significantly lower in CD1d-deficent mice (which lack NKT cells) when compared to WT mice [25]. Although the exact mechanisms for the observed response remain elusive, these results clearly implicate CD1d-restricted NKT cells in an early amplification step of cytokine and chemokine production during the innate response elicited by *T. cruzi *GIPL. Therefore, the *in vivo* effects of *T. cruzi* PAMPs deserve further investigation with regard to their mode of recognition by, and action on, different cell types.

 Infective *T. cruzi* trypomastigotes invade host cells using at least two different strategies, either by an active process recruiting host-cell lysosomes to the area of parasite cell contact or by an alternative pathway, in which the parasite infects phagocytic cells through conventional phagocytosis/endocytosis mechanism [26–29]. While the general current view is that TLRs do not function directly as phagocytic receptors, studies have demonstrated that TLR signaling by means of MyD88 can enhance phagosome acidification and function, the so-called phagosome maturation, which is required for effective sterilization of its contents [30]. In accordance to that, we have demonstrated that the levels of *T. cruzi* internalization by macrophages is not affected in three different TLR4-deficient mouse strains (C3H/HeJ, C57BL/10ScN, and *Tlr4^−/−^*), but TLR4 and parasite colocalize into acidic compartments, and, as soon as 4 h after infection, the percentage of TLR4-deficient macrophages infected with *T. cruzi* is significant higher when compared to WT cells, indicating the existence of an early trypanosomicidal mechanism triggered by TLR4, which was also shown to be dependent on the production of NO and reactive oxygen species (ROS) [31]. On the other hand, it was reported that during the invasion of *T. cruzi*, the activation of the Rab5-dependent phagocytic pathway is regulated by TLR2-dependent signals in macrophages [32]. Still, to our knowledge, there are no other studies on the participation of surface TLR pathways in the entrance of trypomastigotes into the host cells.

 An additional TLR2 agonist with adjuvant properties, the *T. cruzi-*released protein related to thiol-disulfide oxidoreductase family, called Tc52, was also described [33]. Surprisingly, however, despite the known *T. cruzi*-derived TLR2 agonists, no differences in parasitemia or mortality were noted following infection of mice genetically deficient in TLR2 [34]. Intriguingly, although TLR2 expression by macrophages stimulated *in vitro* with trypomastigote-derived GPI anchors appears to be essential for induction of IL-12, TNF-*α* and NO [16], when infected, the TLR2-deficient mice mount a robust proinflammatory cytokine and NO production by spleen cells, as well as higher serum levels of IFN-*γ*, when compared to WT mice [34]. This suggests an immunoregulatory role for TLR2 during the infection, maybe due to the action of TLR2 ligands on Tregs [35].

 Interestingly, more recently, *T. cruzi*-derived nucleic acids have been also shown to act as PAMPs. Genomic DNA, which contains abundant oligodeoxynucleotide unmethylated CpG motifs, and total RNA purified from *T. cruzi* promote host cell activation via TLR9 and TLR7, respectively, stimulating cytokine response from macrophages and dendritic cells (DCs) [36–39]. Also, potential TLR7 ligands as guanosine- or uridine-rich single-strand RNA sequences were found by *in silico* analysis in the predicted parasite transcriptome [39]. Indeed, as discussed below, *Tlr9^−/−^* and *Tlr7^−/−^* mice were shown to be more susceptible than WT mice to infection with the parasite [37, 39].

## 4. Resistance to Infection Conferred by Different TLR Pathways

Directly testing the hypothesis that TLR triggering by the above-described PAMPs is crucial for host resistance against the infection is currently not possible, however, due to the absence of *T. cruzi* strains lacking the expression of any of the above-described TLR agonists. On the other hand, studying the course of infection in mice genetically deficient for different TLR-encoding genes, evaluating mortality, parasitemia, and several parameters of the innate and acquired immune responses have brought additional understanding of the impact of the lack of TLR-mediated recognition of *T. cruzi* for development of host susceptibility to the infection. In this context, the critical involvement of TLRs in the host resistance to *T. cruzi* was firstly highlighted in mice deficient for the MyD88 adaptor molecule, which is the main transducer of multiple TLR-signaling pathways [34]. In fact, *Myd88^−/−^* mice were shown to be highly susceptible to infection and to display lower production of proinflammatory cytokines, including IL-12p40 and IFN-*γ*, from innate immune cells [34]. In accordance, we first reported that C3H/HeJ mice, which express a nonfunctional natural mutant of TLR4, are highly susceptible to infection with *T. cruzi* [17], as evidenced by a higher parasitemia and earlier mortality. However, since classical genetic studies previously established that the resistance to *T. cruzi *is governed by multiple genetic factors, including H-2-linked genes [40, 41], the level of protection given by the TLR4 pathway during the infection of C3H/HeJ mice (whose C3H background is classified as “susceptible”) could not be directly compared to the degree of susceptibility of infected *Myd88^−/−^* mice, which are of the resistant C57BL/6 genetic background. Therefore, we further investigated the impact of TLR4 deficiency in the *Tlr4^−/−^* (B6 background) mice [31]. We demonstrated that TLR4 signaling triggers an important early parasiticidal event against *T. cruzi*, which is dependent on the formation of NO and ROS and that splenocytes of *Tlr4^−/−^* infected mice display lower production of the proinflammatory cytokines IFN-*γ* and TNF-*α*, as well as of NO, when compared to WT B6 mice, what would explain the observed higher parasitemia levels in TLR4-deficient mice [31]. Together these results indicate that TLR4, as previously shown for TLR2 and TLR9, also contributes to resistance during the acute phase of infection in B6 mice. TLR4 deficiency by itself, however, does not lead to an earlier mortality in the B6 background [31].

 An interesting study has demonstrated the involvement of TLR9 in the protection against *T. cruzi* infection [37]. TLR9 is one of the members of the TLR family located at the endolysosomal subcellular compartment and can recognize parasite-derived DNA sequences [38]. More importantly, since *Tlr2^−/−^Tlr9^−/−^* double knockouts display higher parasitemia than the single *Tlr2^−/−^* or* Tlr9^−/−^* mice, this work was the first to demonstrate that TLR2 and TLR9 can cooperate, and/or that a degree of redundancy exist among different TLR family members, in the control of parasite replication. Nevertheless, although attaining parasitemia levels comparable to the observed in the* Myd88^−/−^* strain (which lack multiple TLR signaling), *Tlr2^−/−^Tlr9^−/−^* double deficient mice did not show the acute mortality exhibited by *Myd88^−/−^* mice. This observation suggested that other TLR/IL-1R family members, in addition to TLR2 and TLR9, could be involved in the pathogenesis of *T. cruzi *infection. Furthermore, mice lacking both MyD88 and a second adaptor molecule which acts downstream TLR3 and TLR4, called TRIF, were shown to be even more susceptible than *Myd88^−/−^* mice. Contrary to *Myd88^−/−^,* the *Myd88^−/−^Trif^−/−^* double deficient mice were not able to control parasite levels in the bloodstream and die at an earlier time point after infection [42]. The TRIF-dependent pathway is indispensable for the induction of type 1 IFNs through TLR3 and TLR4, but the role of type 1 IFNs in the resistance to infection with *T. cruzi *is controversial [43, 44]. Curiously, although mice single deficient in TRIF or IFNAR1 (type 1 IFN receptor) were shown to be resistant to the infection with *T. cruzi, Myd88^−/−^ Ifnar1^−/−^* double deficient mice display the same highly susceptible phenotype as *Myd88^−/−^ Trif^−/−^* double deficient strain, pointing to a protective role for IFN-*β* and/or IFN-*α* that, however, only becomes apparent when the Myd88 pathway is absent [42]. Therefore, the high sensitivity demonstrated by the *Myd88^−/−^ Trif^−/−^* double deficient mice to infection is in accordance with a role for TLR4 and/or TLR3 in the response against *T. cruzi*, as these members of the TLR family are the only known to use TRIF as a transducer molecule.

 A very recent work studying *Tlr3^−/−^* mice, however, has not supported any role for TLR3 in promoting control of *T. cruzi* parasitemia or host survival [39]. Yet, the possibility exists that a putative function of TLR3 would only become apparent in the concomitant absence of other TLR-family member with redundant function, by analogy to what was previously observed for TLR2, whose involvement in protection against the parasite was only evident in the double *Tlr2^−/−^Tlr9^−/−^* strain [37]. The article also provided, for the first time, evidences that TLR7 is a critical innate immune receptor involved in the recognition of *T.cruzi *RNA and in host resistance to a protozoan infection [39]. Caetano and collaborators analyzed the course of infection in different mouse strains lacking one or multiple endolysosomal TLRs. First, the authors followed the response to infection in a strain of mice called 3d, which has a loss-of-function point mutation in UNC93B1 (an endoplasmic reticulum (ER) resident protein that mediates the translocation of the nucleotide-sensing TLRs from the ER to the endolysosomes) and, consequently, is unresponsive to TLR3, TLR7 and TLR9 ligands (TLR8 is believed to be biologically inactive in mice). The phenotype of 3d mice was shown to be equivalent of the triple deficient *Tlr3^−/−^Tlr7^−/−^Tlr9^−/−^* strain and was intermediary between *Myd88^−/−^* (highly susceptible) and *Tlr9^−/−^* (moderately susceptible). This result suggested the contribution of TLR7, besides TLR9, for the resistance against *T. cruzi*, since, as mentioned,* Tlr3^−/−^* mice were not susceptible to the infection. In fact, *Tlr7^−/−^* mice were shown to display a degree of susceptibility comparable to *Tlr9^−/−^* mice [39].

 Collectively, to date, the analysis of different mice strains lacking one or multiple TLR pathways demonstrated that TLR2, TLR4, TLR7, and TLR9 play a role in the resistance to infection with *T. cruzi, *with a degree of redundancy between them. The direct comparison between the levels of susceptibility displayed by the diverse TLR-deficient strains of mice is not always possible though, due to the fact that the above-cited studies employed different strains of the parasite, as Y [31, 34, 37], Tulahuén [42], or CL-Brener [39] strains, each of them presenting different virulence, tissue tropism, and time course of parasitemia, and which may also express PAMPs with different fine structures or levels of expression. Nevertheless, important issues have been revealed in those studies concerning the role of TLRs in innate and acquired immunity against *T. cruzi, *as discussed below.

## 5. TLRs in the Innate and Acquired Responses to *T. cruzi*

In the first 7 to 10 days following infection, before acquired immunity is fully activated, innate responses play a key role in containing parasitemia, through the action of microbicidal mediators (reactive nitrogen intermediates—RNI and ROS), whose production is enhanced by the action of proinflammatory cytokines (IL-12, TNF-*α*, and IFN-*γ*) released by macrophages, natural killer (NK), and *γδ* T cells [45, 46]. Then, acquired immunity mediated by the T-helper 1 (Th1) cell response becomes crucial in parasitemia control and host survival. The release of IFN-*γ* by Th1 CD4^+^ cells induces the activation of phagocytic cells for parasite killing. Th1 lymphocytes also provide help for the appropriate production of antibodies (cytophilic and complement-fixing immunoglobulin G2a) and for cytotoxic CD8^+^T cells. The genetic absence, or the experimental blocking, of any of these adaptive responses (antibodies, CD4^+^ or CD8^+^ cells) results in uncontrolled parasite levels and decease [47–49]. Despite the control of parasite burden by different effector responses, however, its elimination is not achieved, leading to chronification of the infection. It is plausible that parasite persistence results from suppression of microbicidal immunity by anti-inflammatory responses mediated by IL-10 and transforming growth factor-*β* (TGF-*β*), as well as by infiltrating myeloid cells with suppressor activity, which succeed and counteract the potent inflammatory response that, otherwise, would lead to life-threatening injury to organs [50, 51].

 It is a current paradigm that the activation of dendritic cells and other innate cells by TLR pathways is required for and play a role in the modulation of acquired responses although the precise function of each member of the TLR family in the responses against *T. cruzi* is still to be fully determined. All the strains of mice with single or multiple TLR deficiency tested to date, which display higher susceptibility to infection with *T. cruzi*, were found to display lower proinflammatory cytokine levels early during infection although the degree of susceptibility varies between the different TLR knockouts, as discussed above. Accordingly, serum levels of IFN-*γ* and IL-12 are low in *MyD88^−/−^* infected mice, as well as the* in vitro* production of IFN-*γ*, IL-12, TNF-*α* and NO by splenocytes obtained from these mice at day 10 postinfection [34]. Similar results were obtained with *Tlr4^−/−^*, *Tlr9^−/−^*, double *Tlr2^−/−^Tlr9^−/−^,* 3d, or *Tlr7^−/−^* mice [31, 37, 39]. These results confirmed others obtained *in vitro,* where lower levels of IL-12 (or NO) and higher number of trypomastigotes were released by splenocytes (or by *in vitro* infected macrophages) of MyD88-, double MyD88/TRIF-, TLR4-, 3d, TLR9-, or TLR7-deficient infected mice [31, 37, 39, 42]. Thus, with the apparent exception of TLR2, several TLRs contribute *in vivo *to the induction of proinflammatory cytokine secretion by infected host cells.

 Beyond TLR's roles in modulation of innate immunity, the current paradigm strongly argues in favor of a critical role of these receptors in shaping the adaptive immune response [9]. This can be achieved mainly by their action on antigen-presenting cells (APCs), either by promoting cross-presentation for CD8 T-cell activation or by increasing the levels of costimulatory molecules and by stimulating the secretion of lineage-specific cytokines as IL-12, IL-6, IL-1*β*, IL-18, and IL-23 by APCs and, thus, promoting Th1 and Th17 differentiation. Although initially controversial, different groups demonstrated the expression of TLRs on activated T cells, as well as the effects of TLR agonists functioning as direct costimulatory signals during the initiation of the adaptive immune response or as an aid in the survival of memory T cells [35]. Therefore, one cannot rule out a direct role for *T. cruzi*-derived PAMPs on T-cell activation and survival during the infection, although to date, evidence favor the hypothesis that the major function of TLRs on T-cell activation during infection is an indirect one.

 Presently, data on the detailed role of TLRs in the activation of acquired immunity during infection with *T. cruzi *are still scarce. Nonetheless, it was first demonstrated that CD4^+^ T cells obtained from infected *Tlr9^−/−^*, *Tlr2^−/−^Tlr9^−/−^,* or *MyD88^−/−^* mice strains produced lower IFN-*γ* when stimulated *in vitro* by infected syngeneic BMDC [37], while CD4^+^ T cells from infected TLR2-deficient mice display levels of IFN-*γ* comparable to WT, as expected due to their resistant phenotype [34]. Of note, the percentage of CD4^+^ IFN-*γ* ^+^ T cells in the spleen of infected *MyD88^−/−^* mice at day 11 and 13 postinfection were shown to be significantly lower compared to WT mice, whereas the percentage of CD4^+^ IFN-*γ* ^+^ T lymphocytes in the spleen of the *Tlr4^−/−^* strain resulted similar to that found in WT mice, in accordance with the relatively higher resistance of this strain, when compared to the other mentioned TLR-deficient mice [31]. Interestingly, the same picture of low CD4^+^ T-cell activation was obtained when analyzing the IFN-*γ* production by CD4^+^ T lymphocytes obtained from infected 3d or *Tlr3,7,9^−/−^* triple deficient mice, even when stimulated *in vitro* with antigen-pulsed WT DCs, suggesting the lower frequency of activated CD4^+^ T cells in infected spleens of these susceptible strains [39]. In the particular case of *MyD88^−/−^* mice, the lower percentage of Th1 cells could also be due to nonresponsiveness to IL-18, since the receptor for this cytokine also relies on MyD88 for signaling, but the fact that mice deficient in IL-18 are not more susceptible to experimental infection with *T. cruzi* [52] argues against this hypothesis. Therefore, in all the TLR-deficient strains tested, susceptibility to infection correlates with lower levels of serum IL-12 and decreased frequency of activated Th1 cells in the spleen.

 A nonexpected result was found, in contrast, when the percentage of CD8^+^ IFN-*γ* ^+^ T cells (measured either by cytometry or by ELISPOT), and the CD8-dependent *in vivo* cytotoxic activity was measured in *Tlr2^−/−^, Tlr4^−/−^, Tlr9^−/−^,* and in *MyD88^−/−^* infected mice, as both parameters were preserved to WT levels in all the above-cited deficient strains [31]. More recently, the maintenance of the frequency of CD8^+^ T cells specific for an immunodominant peptide to WT levels has been also demonstrated in *MyD88^−/−^, Tlr9^−/−^,* and 3d mice [39] although *in vitro* the levels of IFN-*γ* secretion were lower in cell cultures of *MyD88^−/−^* and 3d, but not of* Tlr9^−/−^* mice. A first possible interpretation of these results is that none of the tested TLR pathways is essential for the generation of cytotoxic CD8^+^ T cells during *T. cruzi* infection. Of note, the TLR3- and TLR4-triggered TRIF pathway is preserved in *Myd88^−/−^* mice, hence, their activation would lead to type I IFN secretion and consequent DC maturation, resulting in the normal CD8^+^ T-cell response observed in these mice. Also in accordance with this hypothesis, extensively discussed by us in a previous work [31], doubly MyD88/TRIF-deficient (as MyD88/IFNAR1 DKO) mice are more sensitive to infection and do not control parasitemia [42]. Alternatively, other signaling molecules and innate recognition systems can be involved in the generation of CD8^+^ T-cell responses. For example, it was described that NFATc1 activation and IFN-*γ* production in a TLR-independent pathway may lead to DC maturation during *T. cruzi *infection [53]. Also, DC maturation may be induced by bradykinin B2 receptors (B2Rs) after the release of pro-inflammatory bradykinin peptide by the parasite proteases during infection [54]. Thirdly, a recent work, cited above, has demonstrated the activation of NOD receptors by *T. cruzi* infection [15] though it is still not clear whether these two latter pathways would function independently of TLRs for licensing CD8^+^ T-cell effector functions.

 Therefore, the lower levels of CD4^+^ effectors observed in infected MyD88- or TLR*-*deficient mice seem sufficient for their help to CD8^+^ T cells but might not be enough for inducing the necessary B-cell-mediated response, or for CD8^+^ T lymphocyte mobilization to parasite-infected tissue other than spleen, as heart or liver, for example. Although a recent report that described a role for IL-17A in host protection against acute infection [55] and a role for Th17 cells in regulating parasite-induced myocarditis has been shown during *T. cruzi* infection in mice [56], nothing is known at the present time about the possible modulation of this T helper subset, and its consequences to infection with *T. cruzi*, in the absence of TLR signaling. Undoubtedly, more work is necessary for a full understanding of the effects of *T. cruzi*-induced TLR signaling in the control of adaptive immunity against the parasite.

**Figure 1:**
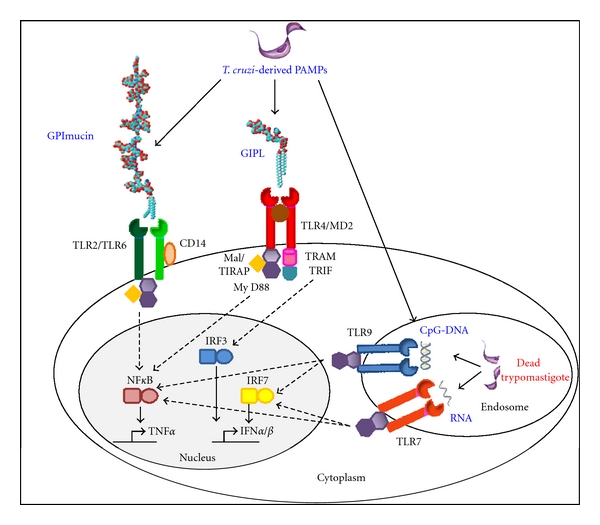
*T. cruzi*-derived PAMPs are recognized by different TLRs. The recognition of different *T. cruzi* molecules, like parasite surface glycoconjugates and nucleic acids, occurs through distinct Toll-like receptors, which are localized at the cellular plasma or endoplasmic membranes, respectively, and are differentially expressed by various innate immune cell types. GPI anchors of mucin-like glycoproteins activate TLR2/TLR6 heterodimer, GIPL is an agonist for TLR4, genomic DNA activates endosomal TLR9, and TLR7 is involved in parasite RNA recognition. TLRs induce NF-*κ*B and/or IRFs activation via their interaction with different TIR domain-containing adaptor molecules. Of these, MyD88 and Mal/TIRAP are required for TLR2 and TLR4 activation of NF-*κ*B. In a MyD88-independent way, TRIF and TRAM signal downstream TLR4, activating IRF3. TLR7 and TLR9 activate NF-*κ*B and IRF7 via MyD88. NF-*κ*B activation leads to proinflammatory cytokines production, such as TNF-*α* and IL-12, whereas IRFs are required for type I IFN gene transcription.

 In summary, the present data support the idea that a degree of redundancy exists among different TLR family members, meaning that each of the TLR pathways may not be individually essential for the resistance to infection. *T. cruzi *displays various ligands for different TLRs (see Figure 1) and only the concomitant absence of signaling through multiple TLR receptors, but not their individual deficiency, results in a high degree of susceptibility to the infection. 

 No discussion about the role of TLRs in the infection by *T. cruzi* could be complete without some speculation concerning the possibility that the immunological response elicited through TLR pathways might have a role in the progression of the disease toward its chronic phase, CCC. Both *T. cruzi*- and heart tissue-specific responses have been put in evidence and may be important for the pathology of CCC although a consensus does not exist about the relative contribution of each of these responses for CCC [57]. Whatever the answer to this question might be, TLR signaling could be implied in the process, since beside their role in the triggering of the adaptive response to pathogens, as above discussed, several studies have also reported the contribution of TLR-family members in the induction of autoimmunity [58]. However, studies on the chronic stage of infection with *T. cruzi* are difficult in mice of C57BL/6 genetic background (as all the available TLR knockout strains), due to the scarcity of good experimental models capable of inducing in these mice the pathophysiologic traits observed in the human condition. Notwithstanding, a study of 169 patients with chronic chagasic cardiomyopathy and 76 *T. cruzi*-infected asymptomatic individuals revealed that *T. cruzi*-infected patients who are heterozygous for the MAL/TIRAP S180L variant (which leads to a decrease in signal transduction upon ligation of TLR2 or TLR4 to their respective ligand) may have a lower risk of developing CCC [59]. Interestingly, it was also demonstrated that TLR2 functions as the main upstream regulator of hypertrophy triggered in isolated murine cardiomyocytes by *T. cruzi* [60]. Therefore, the study of the involvement of TLR signaling in experimental models of the chronic phase of the Chagas' disease could be of considerable value in elucidating the pathophysiology of CCC, which remains one of the major causes of heart failure among younger individuals in Latin America today. Moreover, determining precisely how TLR-TRIF-MyD88 activation could trigger and modulate the immune response against *T. cruzi* will be of critical relevance for vaccine development against this important human parasite.

## 6. Vaccination against *Trypanosoma cruzi* Infectiona

The strong specific immune response developed in most hosts following *T. cruzi* infection does not eliminate the parasite, and parasite persistence is considered to be the main factor contributing to the late symptoms of Chagas disease. Therefore, eliminating the parasite at the early stage (acute phase) prevents parasite survival and may be an interesting route to avoid chronic phase immunopathology. Prophylactic vaccination would help to reduce or completely eliminate the parasite burden and thus represents a desirable method to restrict the development of chronic symptoms of the disease. Until recently, vaccination was not considered a cost-effective measure for containment of the disease transmission because other methods of prevention would be simpler and cheaper. Nevertheless, recent detailed analyses have proved that indeed vaccination can be cost effective in a variety of scenarios including the regions where the prevalence is as low as 1% by using a vaccine which the efficacy was only 25% [61]. Based on that, future research programs should consider these calculations to support this type of biotechnological alternative.

Because CD4^+^ and CD8^+^ T cells are critical mediators of the acquired immune response, over the past 20 years, we and others have tested the hypothesis that non-antibody-mediated cellular immune responses to the antigens expressed in the mammalian forms of the parasite could indeed be used for the purpose of vaccination. Using a mouse model of the disease, we confirmed this hypothesis by inducing protective immunity against *T. cruzi *infection specifically mediated by CD4^+^ Th1 and CD8^+^ Tc1 cells specific for antigens expressed by trypomastigotes and amastigotes of *T. cruzi* (reviewed by [62–64]).

*T. cruzi *antigens recognized by immune sera from immune or infected humans or animals served as the basis for researchers to conduct studies using recombinant proteins. These recombinant proteins included members of the large *trans*-sialidase (TS) surface protein family expressed mainly in the infective trypomastigote and amastigote forms of the parasite. The second group of genes belonged to the family of cysteine-proteases (cruzipain) expressed in all of the different forms of the parasite. Other antigens formed a heterogeneous group including molecules such as the flagellar calcium-binding protein, paraflagellar rod protein-2, LYT-1 antigen, ribosomal protein L7a-like protein, and KMP11, among others (reviewed by [62, 63]).

To induce *T. cruzi*-specific T lymphocytes and protective immunity against an experimental infection, several delivery antigens were used successfully such recombinant proteins mixed in the presence of distinct adjuvants, plasmid DNA, recombinant viruses, and bacteria. Very recently, genetically attenuated parasites have been also successfully generated for the purpose of the development of an oral veterinary vaccine [65]. Protective immune response in the mouse model was measured by the reduction in acute phase parasitemia, tissue parasitism, and mortality. In most cases, immunity elicited by these antigens was associated with type I immune response, generated by IFN-*γ*-producing CD4^+^ and/or CD8^+^ T cells. Some of the mechanisms mediating protective immunity were investigated. Following intranasal immunization with (TS) in the presence of the TLR9 activator CpG ODN, the absence of CD4^+^ or CD8^+^ T cells renders the vaccinated animals completely susceptible to infection. Because these animals were genetically deficient, these cells can be required for the induction or the effector phase of the immune response, or both. Similarly, CD8-deficient mice failed to generate protection after immunization with native Par-2 protein emulsified in CFA or recombinant adenovirus expressing TS or ASP-2 genes [66, 67]. Upon plasmid immunization, the depletion of either CD4^+^ or CD8^+^ T cells completely reversed protective immunity, thus demonstrating a nonoverlapping role for these two subpopulations [68, 69]. Following vaccination with recombinant protein of ASP-2 in alum and CpG ODN, only depletion of CD8^+^, but not CD4^+^, T cells reversed protective immunity [70]. Finally, vaccination with a single *T. cruzi *epitope, recognized by CD8^+^ T cells [71], elicited a protective immune response using a heterologous prime-boost strategy with recombinant adenovirus and vaccinia virus. These experimental systems found type 1 CD4^+^ and CD8^+^ T cells to be necessary, confirming the general paradigm that type 1 CD4^+^ and CD8^+^ T cells do play a key role in protective immunity. In agreement with this hypothesis, recent observations have pointed to IFN-*γ* as a critical mediator of the protective immune response [72]. Also relevant is the fact that protective T cells can be long lived and stable and display a phenotype of effector memory T cells [73, 74]. Another recently added information that might be of general importance for vaccine development has been the fact that the target of these protective CD8^+^ T cells is not only the immune-dominant epitopes, but they can also be subdominant/cryptic T-cell epitopes [75, 76].

The question as to whether other cell types are also critical for the adaptive immunity induced by these recombinant vaccines is currently being investigated. Still, noteworthy is the fact that infection itself elicits strong type 1 immune response, and it is not capable of clearing the parasite completely. This apparent contradiction suggests that there may be qualitative differences between immune responses elicited by infection or vaccination that are not revealed by the analyses of the cytokine pattern. In fact, ongoing experiments strongly argue that there are qualitative differences that account for the protective properties of the T cells expanded after infection in genetically vaccinated mice (Vasconcelos, unpublished results).

In spite of clear evidence that immunization with *T. cruzi *antigens can provide protective immunity as measured by a reduction in acute phase parasitemia, tissue parasitism, and mortality, it is not clear whether immunization will lead to either remission or a cure of the chronic phase symptoms of the disease. To determine the role of immunization in reducing chronic phase disease symptoms, a number of experiments using different animal models must be performed. In many of the models described above, tissue inflammation and parasitism in the late chronic phase were significantly reduced following prophylactic vaccination [69, 77, 78]. Therefore, it is possible that prophylactic vaccinations indeed halt the development of the chronic phase immunopathologies. Nevertheless, the most compelling evidence of a vaccine's ability to reduce the immunopathology was obtained by therapeutic immunization with *T. cruzi *genes encoding the TSA and Tc24 genes [79]. Whether these results are reproducible using different combinations of mouse and parasite strains remains to be seen.

In conclusion, in spite of the pessimism of certain researchers, there are a number of experimental evidences that support the fact that a vaccine against Chagas disease can be obtained for veterinary use. This type of vaccine could have a definitive impact on disease transmission. Whether this knowledge can be translated into a vaccine for a human use will still require considerable body of experimental and clinical studies [80].

## 7. TLRs and the Development of New Adjuvants

Understanding how pathogens initiate and direct immune responses can provide useful perspectives for vaccine development. In fact, in the last twenty years, the increasing knowledge of the cellular and molecular mechanisms by which innate immunity signaling triggers particular responses from APCs has allowed the design of new defined adjuvants. For example, monophosphoryl lipid A (MPL) is a detoxified lipid A derivative of lipopolysaccharide from *Salmonella enterica*, which acts as a TLR4 partial agonist. It preferentially induces the TRAM/TRIF-signaling pathway and, consequently, has lower toxicity when compared to LPS but retains its adjuvant properties [81]. MPL adsorbed to aluminium salts has been used as adjuvant in prophylactic vaccines against different infectious agents, including an antihuman papillomavirus (HPV) vaccine approved in Australia, Europe, and the USA for the prevention of cervical cancer [82]. Therefore, research focused on the identification and characterization of PAMPs from* T. cruzi*, as well as from other pathogens, may provide us with new TLR agonists, which combined to known adjuvant molecules will allow the creation of a new generation of vaccines, which will be able, for example, to direct the immune response toward a dominant Th1 profile (required for protection against intracellular pathogens) and will be endowed with long-lasting immunological memory. TLR agonists may also be employed not only in prophylaxis but also in therapeutic approaches. This fascinating subject is however beyond the scope of the present review and has recently been discussed in detail by other authors [13, 83].
